# Safety risk assessment of subway shield construction under-crossing a river using CFA and FER

**DOI:** 10.3389/fpubh.2024.1279642

**Published:** 2024-02-02

**Authors:** Kuang He, Tianlin Cui, Jianhua Cheng, Yanlong Huang, Hujun Li, Huihua Chen, Ke Yang

**Affiliations:** ^1^Southwest Jiaotong University Rail Transit Transportation System National Primary Laboratory, Chengdu, Sichuan, China; ^2^Zhengzhou Metro Group Co. Ltd., Zhengzhou, Henan, China; ^3^Department of Civil Engineering, Henan Polytechnic University, Jiaozuo, Henan, China; ^4^Department of Civil Engineering, Central South University, Changsha, Hunan, China

**Keywords:** shield construction safety risks assessment, subway under-crossing a river, safety risk list, safety assessment model, confirmatory factor analysis, fuzzy evidence reasoning

## Abstract

Numerous subway projects are planned by China's city governments, and more subways can hardly avoid under-crossing rivers. While often being located in complex natural and social environments, subway shield construction under-crossing a river (SSCUR) is more susceptible to safety accidents, causing substantial casualties, and monetary losses. Therefore, there is an urgent need to investigate safety risks during SSCUR. The paper identified the safety risks during SSCUR by using a literature review and experts' evaluation, proposed a new safety risk assessment model by integrating confirmatory factor analysis (CFA) and fuzzy evidence reasoning (FER), and then selected a project to validate the feasibility of the proposed model. Research results show that (a) a safety risk list of SSCUR was identified, including 5 first-level safety risks and 38 second-level safety risks; (b) the proposed safety risk assessment model can be used to assess the safety risk of SSCUR; (c) safety inspection, safety organization and duty, quicksand layer, and high-pressure phreatic water were the high-level risks, and the onsite total safety risk was at the medium level; (d) management-type safety risks, environment-type safety risks, and personnel-type safety risks have higher expected utility values, and manager-type safety risks were expected have higher risk-utility values when compared to worker-type safety risks. The research can enrich the theoretical knowledge of SSCUR safety risk assessment and provide references to safety managers for conducting scientific and effective safety management on the construction site when a subway crosses under a river.

## 1 Introduction

China, being the world's second-largest economy, is rushing toward becoming an urbanized and industrialized country (1). The government has gradually enacted laws and increased investment to facilitate infrastructure construction, to create jobs, and improve their people's living standards (2–4). The subway, as a typical city infrastructure characterized by high traffic efficiency, safety, stability, and energy-saving, is often found on the city planners' desks (5, 6). The city is a highly complex human settlement system, often with roads, rails, and rivers running through it. Thus, subway construction can hardly avoid crossing a river. Although most of the subway project contractors select shield construction as the tunneling technique considering its high safety and low impact, the construction process is often susceptible to safety accidents (7–9) (e.g., gushing water, collapse) when under-crossing a river because of the complex geological and hydrological environment. These safety accidents often cause substantial casualties and monetary losses (10, 11). For instance, a gushing water accident occurred when Foshan subway line 2 crossed under a river, causing 12 deaths, 8 injuries, and a wide-range road collapse (12). Therefore, there is an urgent need to investigate the safety risks of subway shield construction under-crossing a river (SSCUR).

Large scale studies have been conducted on shield construction safety risk, and their research mainly focused on safety risk identification and safety risk assessment. Regarding safety risk identification, previous scholars have identified shield construction safety risks from different perspectives. For example, Pan et al. (13) followed the framework of “personnel-machine-material-method-environment,” and identified personnel-type, machine-type, environment-type, and management-type safety risks during shield construction; Liu et al. (14) found out the shield construction safety risks by using a questionnaire survey, including tunnel excavation, shield machine launch, segment assembly, special procedures and conditions, shield machine arrival, grouting, lead excavation, slag removal, and shaft construction. As for safety risk assessment, previous researchers often used the analytic hierarchy process (15), cloud model (15), fault tree analysis (16), Bayesian network (17, 18), and backpropagation (BP) neural network (19–21) as assessment techniques. For example, Chung et al. (22) applied a Bayesian network model to evaluate safety risks during tunnel shield construction. A comprehensive analysis of the existing literature found that most studies investigated the shield construction safety risks in construction under-crossing complex geological conditions, and in construction under-crossing existing buildings and pipelines, except for Wu and Zou (21), combined entropy weight method and cloud model to evaluate the static risk of underwater shield tunnel construction. However, only natural environment-type, tunnel-type, and management-type safety risks were discussed in Wu and Zou's study, and the safety risks related to personnel and method, although very important, had not been taken into account. Consequently, the existing safety risk list is incomplete for evaluating the safety risk during SSCUR. Besides, the evaluation approaches mentioned above mainly rely on a small amount of expert data. Thus, how to incorporate more expert data into the evaluation process to obtain more objective risk assessments is also a question that needs further exploration.

To fill the research gaps, the paper examines the safety risk assessment of SSCUR. The main purposes of this study are to: (1) provide a systematic and feasible safety risk list for SSCUR based on literature analysis and experts' evaluation; (2) propose a quantitative method to assess the safety risks of SSCUR; (3) select a case to validate the proposed quantitative approach. The research can enrich the theoretical knowledge of SSCUR safety risk assessment and provide references to safety managers for conducting scientific and effective safety management on the construction site when a subway crosses under a river.

## 2 Literature review

At present, more subway tunnels choose shield construction techniques with the development of shield machine manufacturing technology, because this tunneling technique is characterized by more safety, less environmental impact, and a higher automation level (6, 7). Subway tunnels are generally planned underground; thus, subway tunnel construction often encounters various complex environmental contexts (e.g., under-crossing complex overburden layers, adjacent to rivers, existing pipelines, and tunnels). Previous studies have investigated the shield construction safety risks in some complicated settings, including construction under-crossing the complex overburden layer (6, 23, 24), construction under-crossing existing buildings (25–27), construction under-crossing existing tunnels (28, 29), construction under-crossing existing pipelines (30), and construction adjacent to a bridge (31). The topics of these studies are mainly focused on two aspects, i.e., safety risk identification and safety risk assessment.

### 2.1 Safety risk identification of subway shield construction

Safety risk identification is the prerequisite of the safety risk assessment. The existing literature, project cases, accident reports, and industry standards were often selected as texts for extracting the safety risks, but the previous scholar might select different frameworks to identify safety risks (27, 30). Some scholars used the framework of “machine-environment-management” (Framework 1) (23, 24, 29, 31), and they divided the identified second-level safety risks into machine-type, environment-type, and management-type safety risks. Machine-type safety risks refer to the safety risks arising from machine failure or misoperation; environment-type safety risks refer to the safety risks arising from poor geological and hydrologic conditions; and management-type safety risks focus on the organization or management aspects (e.g., technique arrangements, team or organization management). For instance, Hu et al. (23) used this framework to investigate the subway shield construction safety risks when under-crossing soft overburden layers, and identified geological complex conditions, underground water conditions, the minimum thickness of the overburden layer, the minimum radius of curvature, construction speed, distance from the surrounding environment, and construction management level as second-level safety risks; Zhai et al. (31) applied this view to examine the subway shield construction safety risks when being adjacent to an existing bridge and summed up the relevant second-level safety risks, involving geological and hydrological condition, shield construction parameters, tunnel conditions, bridge conditions, and organization and management risks. Some other scholars argued the subway shield construction is a complex system, and the “personnel-machine-environment” system was important when tackling this type of complex system problem, thus they used the framework of “personnel-machine-environment” (Framework 2). Compared to Framework 1, this framework incorporates the management-type safety risks into environment-type safety risks and highlights personnel's roles in safety risk controlling. Besides, the personnel-type safety risks are only related to the individual participating in construction and were further subdivided into worker-type safety risks and manager-type safety risks (5, 6). For example, Chen et al. (5) and Liu et al. (6) used this framework to examine subway shield construction safety risks when under-crossing complex overburden layers, subdivided environment-type safety risks into natural environment safety risks, manmade environment safety risks, management environment safety risks, and working environment safety risks, and identified 35 relevant second-level safety risks. Other scholars slightly modified Framework 2, they sorted out the management-type safety risks from the environment-type safety risks like Framework 1 and formed the framework of “personnel-machine-environment-management” (Framework 3). For instance, Pan et al. (13) and Wu et al. (32) utilized this framework to investigate the shield construction safety risks respectively. Pan et al.'s research identified 12 second-level safety risks, and Wu et al.'s study listed 15 second-level safety risks. Besides, there exists an all-inclusive framework, i.e., the “personnel-machine-material-method-environment” framework (Framework 4). Compared to the three frameworks mentioned earlier, this framework adds material-type and method-type safety risks. Material-type safety risks refer to the safety risks that arise from raw materials, components, semi-finished products, and finished products during construction (e.g., broken pipe segments, unqualified grouting materials); Method-type safety risks mainly focus on the construction technology aspects (e.g., shield tunneling technology and grouting technology). Li et al. (33) and Fan and Wang (34) examined the subway shield construction safety risk and collected the safety risks based on the framework. Above all, the four frameworks mentioned earlier were aligned with the total quality management theory of “man-machine-material-method-environment.” The differences between these four frameworks stem from the degree of the previous scholars' attention to these different aspects. The four frameworks provide the theoretical or literature basis for the identification framework applied in the study.

### 2.2 Safety risk assessment of subway shield construction

Literature on safety risk assessment focused on designing safety risk assessment models. The safety risk assessment model describes the calculation process of the risk assessment. As there exist multiple safety risks in the index framework, the two key questions in designing the assessment model are the weight-determining method and the safety risk measurement. As for the weight-determining method, earlier research selected the analytic hierarchy process (AHP) to determine the weight system. For instance, Li et al. (33) selected AHP to calculate the weights of safety risks when investigating the safety risks in slurry balancing shield construction. Gradually, some more objective techniques were introduced to mitigate the subjectivity in determining the weights. For instance, Fan and Wang (34) applied ISM-DEMATEL and Shapley value method to determine weights of safety risks to consider the relationships between different safety risks; Zhai et al. (31) selected a combinatorial weighting method by integrating G1 and CRITIC on shield construction safety risk when being adjacent to an existing bridge.

As for the safety risk measurement, previous literature can identify many quantitative methods. The fuzzy comprehensive evaluation method is widely applied in the evaluation of shield construction safety risks (33, 35). For instance, Ren et al. (35) chose this method to assess the total shield construction safety risks when being adjacent to an existing building. The matter-element method is another widely used technique (25, 34). This technique has advantages in determining the risk rating by connecting the risk and its risk criteria (4). At present, the Bayesian network (29, 36), cloud model (24, 32), and evidence reasoning theory (29) were also introduced into the area of safety risks to decrease the impact of uncertainty. Wu et al. (29) integrated fuzzy Bayesian and evidence theory to evaluate the subway shield construction safety risk when under-crossing an existing tunnel; Wu et al. (32) chose the cloud model to evaluate the shield construction safety risks; further, Chen et al. (24) applied the extension cloud theory and optimal cloud entropy to examine the shield construction safety risk when being adjacent to an existing building. Besides, Monte Carlo (31) and Systematic dynamic (SD) (13) were also applied to the shield construction safety risk assessment by simulating the probability sampling process and the dynamic relationships between different safety risks.

The above studies showed that an evaluation approach including a weight-determining method and safety risk measurement method was needed for a multifactor evaluation framework. For the weight-determining method, the key to the method selection is to mitigate the subjectivity caused by fewer experts. Confirmatory factor analysis (CFA) is an effective relationship extraction method based on more data collected by questionnaire (32); thus, the method can significantly reduce the impact of subjective judgment caused by a small number of experts. Therefore, this study applied the CFA to determine the weights of safety risks. Besides, evidence reasoning theory is a useful approach to integrate more information and reduce uncertainty (29), thus we selected fuzzy evidence reasoning to measure the value of a single safety risk and the onsite overall safety risk.

## 3 Safety risk identification of SSCUR

### 3.1 The framework for safety risk identification

**Figure 1:**
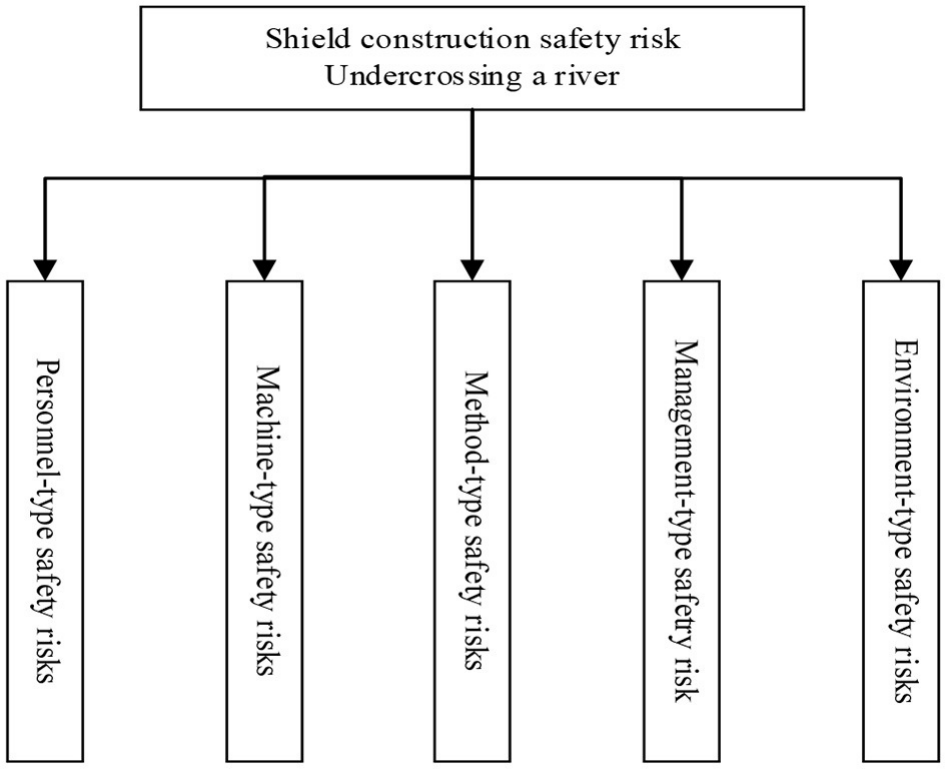
Safety risk identification framework of SSCUR.

Although few studies investigated the safety risks during SSCUR, the frameworks identified in the previous literature can provide feasible frameworks to identify the safety risks. As explained earlier, Framework 3 and Framework 4 are more methodical frameworks. First, Framework 3 does not include material-type safety risks when compared to Framework 4. The reason is that there might be duplication in safety risk statistics in Framework 4, namely, non-standard materials cannot be used in the construction after multiple rounds of material inspections, while the material damage of the final-finished production is mostly caused by the non-standard construction operations, which are analyzed in personnel-type, machine-type, and method-type safety risks (32, 34). Second, Framework 3 distinguishes management-type safety risk from environment-type safety risk. The two are different. Management-type safety risks arise more from organization-level non-compliant behaviors. However, Framework 4 incorporates the method-type safety risk in its analysis views. Therefore, we adjusted Framework 3 based on Framework 4 and proposed the safety risk identification framework for SSCUR. The identification framework is presented in Figure 1. It is noted that personnel-type safety risks arise from inappropriate physical, psychological, and ability conditions, and non-compliant behaviors at the individual level; and the personnel include workers and managers.

### 3.2 The process for safety risk identification

Based on the aforementioned safety risk identification framework, the paper applied a two-step approach to collect the safety risks of SSCUR. First, we used a literature review to identify the related safety risks. Second, we arranged an expert group to evaluate and enrich the pre-identified safety risks.

Step 1. Safety risk identification based on literature review.

We selected CNKI and Scopus as retrieval databases and the searching strategy is ((“safety” OR “risk^*^”) AND “shield construction”) OR [“shield construction” AND “river^*^” AND (“safety” OR “river”)]. We also limited the type of literature to articles and reviews. The search shows 84 English articles and 75 Chinese articles. The researchers selected the final relevant studies by reading titles, abstracts, and texts. Two selection criteria were used to determine the relevance to the research, including: (a) removing the studies which are unrelated to shield construction, for example, the term “shield construction” and “safety” are all in the abstract of Yang et al.'s study (37), but this research is focused on the on-site shield spoil utilization; (b) removing the studies which are unrelated to shield construction safety or shield construction risks, for instance, Dai et al.'s (38) paper mentioned “shield construction” and “safety,” but the research is about the attitude and position prediction of shield machine in tunneling. After the exclusion process, 54 articles were retained, including 31 English articles and 23 Chinese articles. Safety risks were initially extracted from the retained articles based on the identification framework mentioned earlier. The preliminary safety risk list (including first-level safety risk categories and second-level safety risks) is presented in Appendix A.

Step 2. Safety risk evaluation and enrichment based on experts' group evaluation.

Twenty safety management experts were invited to evaluate the safety risk list gained in the previous step (see Appendix A). The invited 20 experts all have at least 5 years of construction experience in shield construction, and they include 2 professor-level experts, 5 senior engineers, and 13 site engineers. The experts were asked to mark whether the safety risks in the list were important or not and to propose any improvements to the safety risk list. If more than 10 experts marked the safety risk, then the safety risk was retained in the list. Based on the statistics on the experts' marks, safety control, failure of soil transport vehicle, safety management system, and high underground water were removed from the safety risk list. Besides, 5 experts were suggesting that incorporate safety skills and safety experience into safety competency. After the above-mentioned amendment, the final safety risk list of SSCUR was presented in Table 1.

**Table 1:** Safety risk list of SSCUR.

**First-level safety risks**		**Second-level safety risks**
Personnel-type safety risks	Worker-type safety risks	W1: Physical and psychological health; W2: Safety awareness; W3: safety competency.
	Manager-type safety risks	M1: Safety management awareness; M2: Safety management competency; M3: Safety management intention; M4: Safety communication; M5: Safety inspection.
Machine-type safety risks		MA1: Failure of cutter head system; MA2: Failure of thrust cylinder system; MA3: Failure of screw conveyor; MA4: Failure of segment erector; MA5: Failure of grouting system; MA6: Failure of ventilation system; MA7: Failure of electrical equipment.
Method-type safety risks		ME1: Improper bank reinforcement program; ME2: Inadequate geological and hydrological investigation; ME3: Improper construction monitoring program; ME4: Improper excavation and incremental launching program; ME5: Improper center line control program; ME6: Improper soil conditioning program; ME7: Improper grouting program; ME8: Sealed water-proof program; ME9: Improper contingency plan.
Management-type safety risks		MN1: Safety culture; MN2: Safety institution; MN3: Safety organization & duty; MN4: Safety training & education.
Environment-type safety risks	Geological environment safety risks	GE1: Levee; GE2: Spur dike; GE3: Shallow overburden layer; GE4: Quick sand layer; GE5: Hydrogeological exploration borehole; GE6: Subterranean boulders; GE7: Subterranean voids.
	Hydrological environment safety risks	HE1: High-pressure phreatic water; HE2: High-pressure pietistic water.
	Gaseous environment safety risks	GA1: Marsh gas (methane, hydrogen sulfide, etc.)

## 4 Safety risk assessment model for SSCUR

We designed a safety risk assessment model for SSCUR. The assessment model integrated the confirmatory factor analysis (CFA) (39, 40) and the fuzzy evidence reasoning (FER) (41, 42). The CFA method was utilized to determine the weights of the safety risks, and the FER method was applied to measure the single safety risk or the onsite total safety risk.

### 4.1 Weights calculation based on CFA

Confirmatory factor analysis (CFA) is a widely used data analysis method belonging to factor analysis. Compared to the exploratory factor analysis, which is used to find out the common factor structure from the messy data, CFA aims at validating the feasibility of pre-identified common factor structure (i.e., dimension structure) (43, 44).

A questionnaire survey was chosen to collect data for further CFA (45). The questionnaire is presented in Appendix B. The questionnaire survey was conducted by using the Wenjuanxing platform (46) and the compiled online questionnaire was sent to onsite managers who have cooperation relationships with the researchers. A total of 232 questionnaires were distributed and 197 responses were retained after the validity test. The demographic information of 197 respondents was presented in Appendix C.

The retained data were first imported into SPSS 23 to test the reliability (47, 48). Cronbach's alpha was 0.942 (49), indicating the collected data have high internal consistency and reliability. Bartlett's test of sphericity produced a significant *p*-value (*p* < 0.001), showing that the data follow the normal distributions (50). The value of KMO was 0.921, suggesting the high correlations between the items and good sampling adequacy for factor analysis (39, 51).

**Figure 2:**
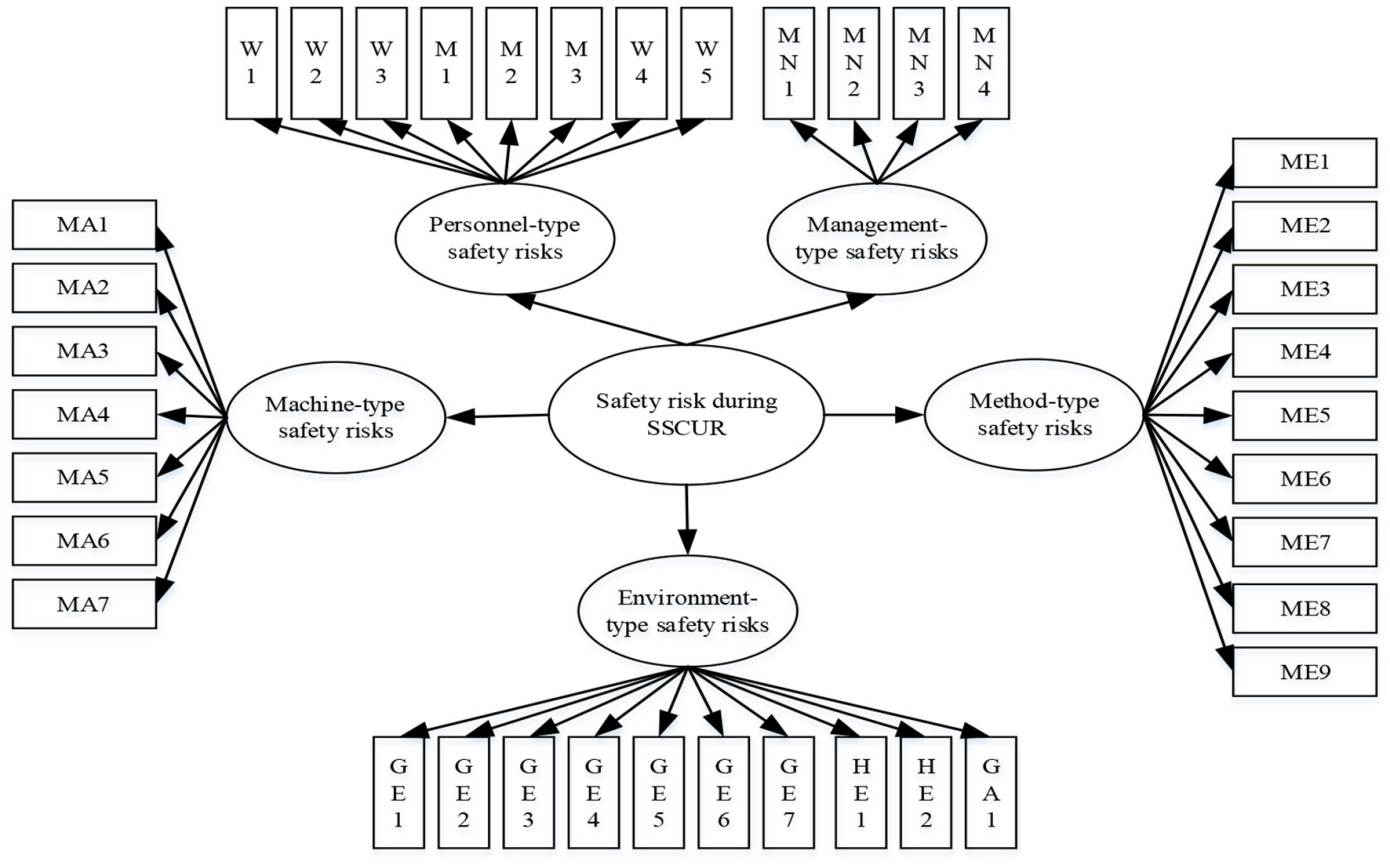
Safety risk identification framework of SSCUR.

Then, the collected data were imported into AMOS 23 to conduct a CFA (52, 53). The concept model was constructed based on the safety risk framework and it was presented in Figure 2. CFA shows a better fit (χ2/df = 2.33 < 3; CFI = 0.903) and the standard path coefficients can be gained, which are shown in Table 2. The standard path coefficients denote the relationship strength between different variables (54–57), namely the second-level safety risks and their first-level safety risk. Thus, based on the standard path coefficients, we calculated the weights of the safety risks, which are shown in Table 3.

**Table 2:** Standard path coefficients of the CFA.

**Relationship path**	**SPC**	**Relationship path**	**SPC**	**Relationship path**	**SPC**
PTSR → W1	0.592	MATSR → MA4	0.682	ETSR → GE3	0.642
PTSR → W2	0.712	MATSR → MA5	0.769	ETSR → GE4	0.659
PTSR → W3	0.607	MATSR → MA6	0.622	ETSR → GE5	0.681
PTSR → M1	0.406	MATSR → MA7	0.625	ETSR → GE6	0.588
PTSR → M2	0.570	METSR → ME1	0.555	ETSR → GE7	0.716
PTSR → M3	0.739	METSR → ME2	0.583	ETSR → HE1	0.755
PTSR → M4	0.711	METSR → ME3	0.686	ETSR → HE2	0.757
PTSR → M5	0.729	METSR → ME4	0.698	ETSR → GA1	0.694
MNTSR → MN1	0.730	METSR → ME5	0.532	SRSSCUR → PTSR	0.737
MNTSR → MN2	0.838	METSR → ME6	0.629	SRSSCUR → MNTSR	0.785
MNTSR → MN3	0.802	METSR → ME7	0.777	SRSSCUR → MATSR	0.576
MNTSR → MN4	0.762	METSR → ME8	0.753	SRSSCUR → METSR	0.464
MATSR → MA1	0.677	METSR → ME9	0.698	SRSSCUR → ETSR	0.948
MATSR → MA2	0.495	ETSR → GE1	0.695	-	-
MATSR → MA3	0.596	ETSR → GE2	0.612	-	-

SPC, standard path coefficient; PTSR, personnel-type safety risks; MNTSR, management-type safety risks; MATSR, machine-type safety risks; METSR, method-type safety risks; ETSR, environment-type safety risks; SRSSCUR, safety risk of SSCUR.

**Table 3:** Standardized weights of the safety risks.

**Safety risks**	**Weights**	**Safety risks**	**Weights**	**Safety risks**	**Weights**
W1	0.117	MA4	0.153	GE3	0.094
W2	0.140	MA5	0.172	GE4	0.097
W3	0.120	MA6	0.139	GE5	0.100
M1	0.080	MA7	0.140	GE6	0.086
M2	0.113	ME1	0.094	GE7	0.105
M3	0.146	ME2	0.097	HE1	0.111
M4	0.140	ME3	0.116	HE2	0.111
M5	0.144	ME4	0.118	GA1	0.102
MN1	0.233	ME5	0.090	PTSR	0.208
MN2	0.268	ME6	0.106	MNTSR	0.223
MN3	0.256	ME7	0.131	MATSR	0.133
MN4	0.243	ME8	0.127	METSR	0.163
MA1	0.152	ME9	0.118	ETSR	0.272
MA2	0.111	GE1	0.102	-	-
MA3	0.133	GE2	0.090	-	-

The weight of a safety risk was calculated by using the SPC value of the safety risk dividing the total SPC values of the safety risks under the same upper-level safety risk category.

### 4.2 Safety risk measure based on FER

The FER algorithm includes the follow-up five steps.

Step 1. Using triangular fuzzy numbers to represent safety risks.

Safety risk R can be expressed as R = P × S, that is, the production of the occurrence probability of a safety risk P and the consequence severity of a safety risk S. Due to the influence of uncertain factors, it is often difficult to measure the safety risks quantitatively. A practical and effective approach is to apply qualitative descriptions to represent safety risk grades (41). The occurrence probability of a safety risk can be qualitatively expressed on a verbal scale as “extremely low,” “low,” “relatively high,” “high,” and “extremely high.” The consequence severity can also be described as “no impact,” “minor,” “large,” “dangerous,” and “catastrophic.” In this paper, the verbal assessment grade descriptions of P and S are transformed into triangular fuzzy numbers, and the corresponding relationships were shown in Table 4.

**Table 4:** Corresponding relationships between verbal assessment grades and the triangular fuzzy numbers.

**Grade**	**Occurrence probability**	**Consequence severity**	**The triangular fuzzy numbers**
1	Extremely low	No impact	(0.00, 0.00, 0.25)
2	low	Minor impact	(0.00, 0.25, 0.50)
3	Relatively high	Large impact	(0.25, 0.50, 0.75)
4	High	Dangerous	(0.50, 0.75, 1.00)
5	Extremely high	Catastrophic	(0.75, 1.00, 1.00)

Assuming that the occurrence probability *P* and the consequence severity *S* are represented by triangular fuzzy numbers, i.e., $\overset{\sim }{P}=\left(l_{P},m_{P},u_{P}\right)$, $\overset{\sim }{S}=\left(l_{S},m_{S},u_{S}\right)$, then the value of the corresponding safety risk is also a triangular fuzzy number, which can be represented by Equation 1 (58, 59).


(1) $$\begin{array}{l} \overset{\sim }{R}=\left(l_{P}l_{S},m_{P}m_{S},u_{P}u_{S}\right) \end{array}$$

Step 2. Constructing the fuzzy confidence structure model for the safety risks assessment grades.

Assuming that there exist N assessment grades for each safety risk and the corresponding membership functions are known, thus we can establish the fuzzy confidence structure of the safety risk assessment grades, which is presented by Equation 2.


(2) $$\begin{array}{l} FBS\left(R\right)=\left\{\left(FH^{n},\beta^{n}\right),n=1,2,3,\cdots \;,N\right\} \end{array}$$

In the Equation 2, *FH*_*n*_ denotes the fuzzy assessment grades; N denotes the numbers of the assessment grades; β_*n*_ denotes the confidence level of R at fuzzy assessment grades, besides, β_*n*_≥0 and $\overset{N}{\underset{n=1}{\sum }}\beta_{n}\leq \;1$.

**Figure 3:**
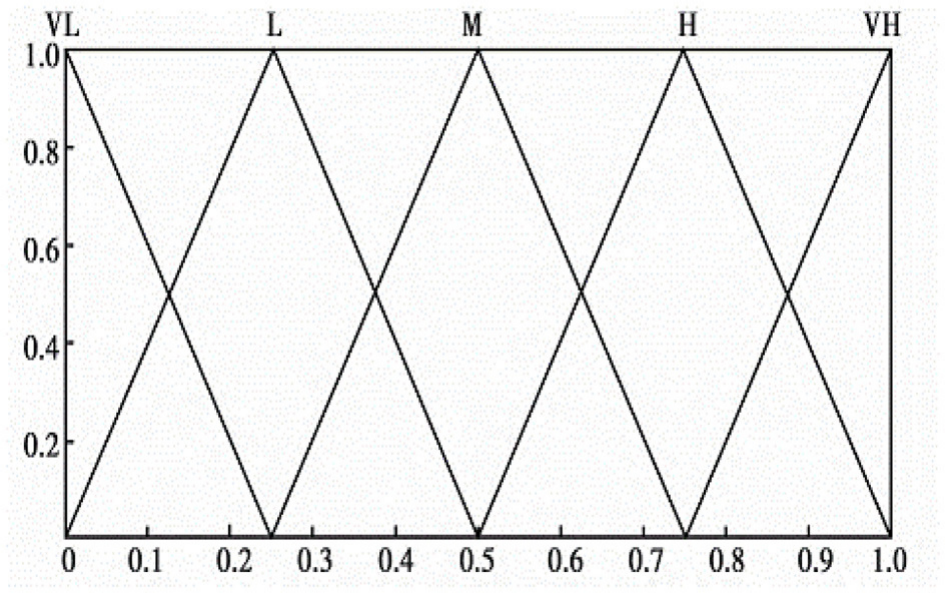
The membership functions of the 5 assessment grades.

In this article, we assumed that there exist five assessment grades for a safety risk and the corresponding membership functions adopted fuzzy triangular numbers. The definition and description of the safety risks grades are presented in Table 5. As such, the fuzzy confidence structure of the safety risks assessment grades can be expressed by Equation 3 and the membership functions of the 5 assessment grades are depicted in Figure 3.


(3) $$\begin{array}{l} FBS\left(R\right)=\left\{\left(FH^{n},\beta^{n}\right),n=1,2,3,4,5\right\} \end{array}$$

Step 3. Calculating the confidence level of each safety risk.

**Table 5:** Definition of safety risk assessment grades and their membership functions.

**Num**.	**Level of safety risk**	**Definition**	**The membership functions**
1	Extremely low (EL)	The safety risk is acceptable	(0.00, 0.00, 0.25)
2	Low (L)	The safety risk is acceptable, and if the safety risk cost is acceptable, measures should be taken to reduce the risk.	(0.00, 0.25, 0.50)
3	Medium (M)	If technology is feasible, measures must be taken to reduce the risk.	(0.25, 0.50, 0.75)
4	High (H)	Measures must be taken to reduce the risk.	(0.50, 0.75, 1.00)
5	Extremely high (EH)	Measures must be taken to reduce and control the risk.	(0.75, 1.00, 1.00)

Through this step, we can calculate the confidence structure of each safety risk (β_*n*_) based on the triangular fuzzy values of each safety risk ($\overset{\sim }{R}$). The calculating method includes the four rules below.

(1) Draw the membership curve based on $\overset{\sim }{R}$;

(2) Find out the intersection points between the membership curve of $\overset{\sim }{R}$ and the five safety risk grade membership curves in Figure 3, and calculate the corresponding ordinate of each intersection point (i.e., confidence value of the corresponding assessment grades). If there exist no intersection points, the confidence value on this level is zero.

(3) If there exist 2 intersection points between the membership curve of $\overset{\sim }{R}$ and the five-grade membership curves, the larger ordinate will be used as confidence value;

(4) Sequentially determine the confidence values of a safety risk at the 5 assessment grades, and then standardize the five confidence values. As such the confidence structure of a safety risk *Z*(*R*) can be gained, which is expressed by Equation (4).


(4) $$\begin{array}{l} Z\left(R\right)=\left\{\beta_{EL},\beta_{L},\beta_{M},\beta_{H},\beta_{EH}\right\} \end{array}$$

Step 4. Calculating onsite total safety risk based on evidence reasoning.

Assuming that there exists a risk assessment problem RE with L risk indexes *r*_*i*_ (i = 1, 2, …, L), the weights of these indexes *r*_*i*_ are ω_*i*_, and every risk index follows the fuzzy confidential (Equation 8) (60).


(5) $$\begin{array}{l} m_{i}^{n}=\omega_{i}\beta_{i}^{n},n=1,2,3,\cdots \;,N;i=1,2,3,\cdots \;,L \end{array}$$

(6) $$\begin{array}{l} m_{i}^{H}=1-\overset{N}{\underset{n=1}{\sum }}m_{i}^{n},i=1,2,3,\cdots \;,L \end{array}$$

(7) $$\begin{array}{l} {\bar{m}}_{i}^{H}=1-\omega_{i},i=1,2,3,\cdots \;,L \end{array}$$

(8) $$\begin{array}{l} {\overset{\sim }{m}}_{i}^{H}=\omega_{i}\left(1-\overset{N}{\underset{n=1}{\sum }}\beta_{i}^{n}\right),i=1,2,3,\cdots \;,L \end{array}$$

In the above Equations, $m_{i}^{n}$ denotes the basic fuzzy confidence value of risk index *r*_*i*_ at the fuzzy risk level of *FH*_*n*_, $m_{i}^{H}$ denotes the uncertain risks due to lack of information, which includes ${\bar{m}}_{i}^{H}$and ${\overset{\sim }{m}}_{i}^{H}$.

Let $m_{I\left(i\right)}^{n}$represents the confidence values to the nth assessment grade of an upper-level risk index which the first *I* lower-level indexes support; and $m_{I\left(i\right)}^{H}$ represents the retained probability after all the first *i* lower-level risk indexes are assigned to all the assessment grades, thus the recursive Equations of $m_{I\left(i\right)}^{n}$ and $m_{I\left(i\right)}^{H}$ can be presented in Equations 9–12 (60, 61).


(9) $$m_{I\left(i+1\right)}^{n}=K_{I\left(i+1\right)}(m_{I\left(i\right)}^{n}m_{i+1}^{n}+m_{I\left(i\right)}^{n}m_{i+1}^{H}+m_{I\left(i\right)}^{H}m_{i+1}^{n}$$

(10) $${\tilde{m}}_{I\left(i+1\right)}^{n}=K_{I\left(i+1\right)}({\tilde{m}}_{I\left(i\right)}^{H}{\tilde{m}}_{i+1}^{H}+{\tilde{m}}_{I\left(i\right)}^{H}{\bar{m}}_{i+1}^{H}+{\bar{m}}_{I\left(i\right)}^{H}{\tilde{m}}_{i+1}^{H}$$

(11) $${\bar{m}}_{I\left(i+1\right)}^{n}=K_{I\left(i+1\right)}({\bar{m}}_{I\left(i\right)}^{H}{\bar{m}}_{i+1}^{H}$$

(12) $$K_{I\left(i+1\right)}=(1-\sum_{n=1}^{N}\sum_{\begin{array}{c} t=1\\ t\neq n \end{array}}^{N}m_{I\left(i\right)}^{n}m_{i+1}^{t}{\;}^{-1},i=1,2,3,\cdots ,L-1$$

Then, the fuzzy confidence values of an upper-level risk index β^*n*^ can be gained based on Equation 13.


(13) $$\begin{array}{l} \beta^{n}=\frac{m_{I\left(L\right)}^{n}}{1-{\bar{m}}_{I\left(L\right)}^{H}},n=1,2,3,\cdots \;,N \end{array}$$

Based on the aforementioned processes, we can calculate the fuzzy confidence values of all the second-level safety risks and the onsite total safety risks.

Step 5. Calculating the expected utility values of the safety risks.

Let *u*(*H*_*n*_) represent the expected utility values of the different safety risk grades, then the total expected utility value of safety risks can be gained based on Equation 14 (59).


(14) $$\begin{array}{l} u\left(R\right)=\overset{N}{\underset{n=1}{\sum }}\beta_{n}u\left(H_{n}\right) \end{array}$$

In Equation 14, $u\left(H_{n}\right)=\frac{n}{5},n=1,2,3,\cdots N.$

Step 6. Determining the grades of the safety risks.

If the total expected utility value *u*(*R*)∈, *n* = 1, 2, 3, ⋯ , *N*, then the safety risk grade is *n*.

## 5 Case validation

### 5.1 Project overview

The subway interval between Xizhou Station and South Huanghe Road Station of Zhengzhou Subway line 12 crossed under the Qili River at K12 + 180~K12 + 270. The right line crosses under the Qili River approximately orthogonally, and the left line crosses under the Qili River at an angle of 80°. Currently, the cross-section of the Qili River is a compound cross-section, with an opening width of about 90m and a bottom width of about 50m. Two levels of slope protection are provided on both banks, with a main channel slope of 1:3 and an embankment slope of 1:3. The slope height is 6.4–7.0m. The river bottom is protected by dry masonry, while the first level slope is protected by a C20 concrete slab. The minimum vertical clear distance between the tunnel arch and the river bottom is approximately 18.2m. The section under-crossing is located in the quicksand layer and the underground water is located above the tunnel top.

### 5.2 Identify and assess the safety risks of the project based on experts' group evaluation

The tunnel section under-crossing the Qili River is a highly risky section of the subway interval between Xizhou Station and South Huanghe Road Station. Before under-crossing the Qili River, the project management department invited an experts' group with 15 experts. These experts are from the expert team mentioned earlier (20 safety management experts selected for evaluating safety risks in Section 3.2) and they include 1 professor-level expert, 5 senior engineers, and 9 site engineers. The experts were asked to identify the safety risks while under-crossing the Qili River and then evaluate the occurrence probability and consequence severity of the identified safety risks.

After the onsite investigation, the experts analyzed the project documents and inquired about some project issues from the project managers. Then, a separate safety risk checklist (see Appendix D) was distributed to every expert. The safety risk checklist covers all the pre-identified safety risks in Table 1. The experts marked the safety risks they consider significant and the corresponding occurrence probability and consequence severity of these safety risks. Onsite management personnel collected all the checklists and calculated marked frequency, occurrence probability, and consequence severity of the second-level safety risks. If the marked frequency of a second-level safety risk is less than five, the second-level safety risk is removed. The level of the occurrence probability and consequence severity of a second-level safety risk were determined by the levels selected by most experts. According to the calculation, the selected safety risks by the experts' group were presented in Table 6, and the grades of the occurrence probability and consequence severity of the safety risks were presented in Table 7.

**Table 6:** The identified safety risks by the experts.

**First-level safety risks**		**Second-level safety risks**
Personnel-type safety risk	Worker-type safety risk	W2: Safety awareness; W3: safety competency.
	Manager-type safety risk	M2: Safety management competency; M4: Safety communication; M5: Safety inspection.
Machine-type safety risk		MA3: Failure of screw conveyor; MA4: Failure of segment erector; MA5: Failure of grouting system: MA6: Failure of ventilation system; MA7: Failure of electrical equipment.
Method-type safety risk		ME2: Inadequate geological and hydrological investigation; ME3: Improper construction monitoring program; ME4: Improper excavation and incremental launching program; ME6: Improper soil conditioning program; ME7: Improper grouting program; ME8: Sealed water-proof program;
Management-type safety risk		MN2: Safety institution; MN3: Safety organization & duty; MN4: Safety training & education.
Environment-type safety risk	Geological environment safety risk	GE1: Levee; GE4: Quick sand layer; GE5: Hydrogeological exploration borehole; GE6: Subterranean boulders;
	Hydrological environment safety risk	HE1: High-pressure phreatic water;
	Gaseous environment safety risk	GA1: Marsh gas

**Table 7:** The levels of occurrence probability and consequence severity of the safety risks based on the experts.

**Safety risks**	**Occurrence probability level**	**Consequences severity level**
W2: Safety awareness	3 (Relatively high)	4 (Dangerous)
W3: safety competency	3 (Relatively high)	3 (Large impact)
M2: Safety management competency	3 (Relatively high)	3 (Large impact)
M4: Safety communication	3 (Relatively high)	5 (Catastrophic)
M5: Safety inspection	4 (high)	4 (Dangerous)
MA3: Failure of screw conveyor	3 (Relatively high)	4 (Dangerous)
MA4: Failure of segment erector	2 (Low)	5 (Catastrophic)
MA5: Failure of grouting system	3 (Relatively high)	5 (Catastrophic)
MA6: Failure of ventilation system	3 (Relatively high)	4 (Dangerous)
MA7: Failure of electrical equipment	2 (Low)	5 (Catastrophic)
ME2: Inadequate geological and hydrological investigation	2 (Low)	4 (Dangerous)
ME3: Improper construction monitoring program	3 (Relatively high)	4 (Dangerous)
ME4: Improper excavation and incremental launching program	3 (Relatively high)	3 (Large impact)
ME6: Improper soil conditioning program	3 (Relatively high)	4 (Dangerous)
ME7: Improper grouting program	3 (Relatively high)	5 (Catastrophic)
ME8: Sealed water-proof program	3 (Relatively high)	5 (Catastrophic)
MN2: Safety institution;	3 (Relatively high)	4 (Dangerous)
MN3: Safety organization & duty	4 (High)	4 (Dangerous)
MN4: Safety training & education	3 (Relatively high)	3 (Large impact)
GE1: Levee;	3 (Relatively high)	4 (Dangerous)
GE4: Quicksand layer	4 (High)	4(Dangerous)
GE5: Hydrogeological exploration borehole	3 (Relatively high)	4(Dangerous)
GE6: Subterranean boulders	3 (Relatively high)	3 (Large impact)
HE1: High-pressure phreatic water	4 (High)	4 (Dangerous)
GA1: Marsh gas	3 (Relatively high)	3 (Large impact)

### 5.3 Calculate the values of safety risks based on CFA and ER

According to the transformation rule (see Table 4), the triangular fuzzy numbers of the safety risks can be calculated and the results are presented in Table 8. Following the transformation rules in step 3, we computed the confidence structure of the second-level safety risks, and the results are shown in Table 9.

**Table 8:** The triangular fuzzy values of the safety risks.

**Safety risks**	**Fuzzy occurrence probability**	**Fuzzy consequences severity level**	**Fuzzy values of safety risks**
W2: Safety awareness	(0.25, 0.5, 0.75)	(0.50, 0.75, 1.00)	(0.125, 0.375, 0.750)
W3: Safety competency	(0.25, 0.5, 0.75)	(0.25, 0.5, 0.75)	(0.063, 0.250, 0.563)
M2: Safety management competency	(0.25, 0.5, 0.75)	(0.25, 0.5, 0.75)	(0.063, 0.250, 0.563)
M4: Safety communication	(0.25, 0.5, 0.75)	(0.75, 1.00, 1.00)	(0.188, 0.500, 0.750)
M5: Safety inspection	(0.50, 0.75, 1.00)	(0.50, 0.75, 1.00)	(0.250, 0.563, 1.000)
MA3: Failure of screw conveyor	(0.25, 0.5, 0.75)	(0.50, 0.75, 1.00)	(0.125, 0.375, 0.750)
MA4: Failure of segment erector	(0.00, 0.25, 0.50)	(0.75, 1.00, 1.00)	(0.000, 0.250, 0.500)
MA5: Failure of grouting system	(0.25, 0.5, 0.75)	(0.75, 1.00, 1.00)	(0.188, 0.500, 0.750)
MA6: Failure of ventilation system	(0.25, 0.5, 0.75)	(0.50, 0.75, 1.00)	(0.125, 0.375, 0.750)
MA7: Failure of electrical equipment	(0.00, 0.25, 0.50)	(0.75, 1.00, 1.00)	(0.000, 0.250, 0.500)
ME2: Inadequate geological and hydrological investigation	(0.00, 0.25, 0.50)	(0.50, 0.75, 1.00)	(0.000, 0.188, 0.500)
ME3: Improper construction monitoring program	(0.25, 0.5, 0.75)	(0.50, 0.75, 1.00)	(0.125, 0.375, 0.750)
ME4: Improper excavation and incremental launching program	(0.25, 0.5, 0.75)	(0.25, 0.5, 0.75)	(0.063, 0.250, 0.563)
ME6: Improper soil conditioning program	(0.25, 0.5, 0.75)	(0.50, 0.75, 1.00)	(0.125, 0.375, 0.750)
ME7: Improper grouting program	(0.25, 0.5, 0.75)	(0.75, 1.00, 1.00)	(0.188, 0.500, 0.750)
ME8: Sealed water-proof program	(0.25, 0.5, 0.75)	(0.75, 1.00, 1.00)	(0.188, 0.500, 0.750)
MN2: Safety institution	(0.25, 0.5, 0.75)	(0.50, 0.75, 1.00)	(0.125, 0.375, 0.750)
MN3: Safety organization & duty	(0.50, 0.75, 1.00)	(0.50, 0.75, 1.00)	(0.250, 0.563, 1.000)
MN4: Safety training & education	(0.25, 0.5, 0.75)	(0.25, 0.5, 0.75)	(0.063, 0.250, 0.563)
GE1: Levee	(0.25, 0.5, 0.75)	(0.50, 0.75, 1.00)	(0.125, 0.375, 0.750)
GE4: Quick sand layer	(0.50, 0.75, 1.00)	(0.50, 0.75, 1.00)	(0.250, 0.563, 1.000)
GE5: Hydrogeological exploration borehole	(0.25, 0.5, 0.75)	(0.50, 0.75, 1.00)	(0.125, 0.375, 0.750)
GE6: Subterranean boulders	(0.25, 0.5, 0.75)	(0.25, 0.5, 0.75)	(0.063, 0.250, 0.563)
HE1: High-pressure phreatic water	4 (High)	(0.50, 0.75, 1.00)	(0.250, 0.563, 1.000)
GA1: Marsh gas	(0.25, 0.5, 0.75)	(0.25, 0.5, 0.75)	(0.063, 0.250, 0.563)

**Table 9:** The confidence structure of the safety risks.

**Safety risks**	**Safety risks grades**				
	**EL**	**L**	**M**	**H**	**EH**
Personnel-type safety risk	0.000	0.460	0.536	0.004	0.000
Worker-type safety risk	0.080	0.557	0.330	0.033	0.000
Manager-type safety risk	0.000	0.318	0.630	0.052	0.000
Machine-type safety risk	0.004	0.657	0.340	0.000	0.000
Method-type safety risk	0.001	0.497	0.502	0.000	0.000
Management-type safety risk	0.000	0.437	0.520	0.043	0.000
Environment-type safety risk	0.000	0.420	0.576	0.004	0.000
Geological environment safety risk	0.000	0.434	0.543	0.023	0.000
The total onsite safety risk	0.000	0.495	0.505	0.000	0.000

Then the confidence structure of the first-level safety risks and the onsite total safety risk can be calculated by using Equations 5–14. The results are shown in Table 10.

**Table 10:** The confidence structure of the first-level safety risks and the total onsite safety risk.

**Safety risks**	**Safety risks grades**				
	**EL**	**L**	**M**	**H**	**EH**
Personnel-type safety risk	0.000	0.460	0.536	0.004	0.000
Worker-type safety risk	0.080	0.557	0.330	0.033	0.000
Manager-type safety risk	0.000	0.318	0.630	0.052	0.000
Machine-type safety risk	0.004	0.657	0.340	0.000	0.000
Method-type safety risk	0.001	0.497	0.502	0.000	0.000
Management-type safety risk	0.000	0.437	0.520	0.043	0.000
Environment-type safety risk	0.000	0.420	0.576	0.004	0.000
Geological environment safety risk	0.000	0.434	0.543	0.023	0.000
The total onsite safety risk	0.000	0.495	0.505	0.000	0.000

We determined the risk grades of the safety risks by using Equation 14, and the results were presented in Table 11.

**Table 11:** The risk grades of all the safety risks.

**Safety risks**	**Expected Utility value**	**Grade**	**Safety risks**	**Expected Utility value**	**Grade**
W2: Safety awareness	0.523	M	MN3: Safety organization & duty	0.687	H
W3: safety competency	0.431	M	MN4: Safety training & education	0.431	M
M2: Safety management competency	0.431	M	GE1: Levee;	0.523	M
M4: Safety communication	0.575	M	GE4: Quicksand layer	0.687	H
M5: Safety inspection	0.687	H	GE5: Hydrogeological exploration borehole	0.523	M
MA3: Failure of screw conveyor	0.523	M	GE6: Subterranean boulders	0.431	M
MA4: Failure of segment erector	0.400	M	HE1: High-pressure phreatic water	0.687	H
MA5: Failure of grouting system	0.575	M	GA1: Marsh gas	0.431	M
MA6: Failure of ventilation system	0.523	M	Personnel-type safety risk	0.509	M
MA7: Failure of electrical equipment	0.400	M	Worker-type safety risk	0.463	M
ME2: Inadequate geological and hydrological investigation	0.387	L	Manager-type safety risk	0.547	M
ME3: Improper construction monitoring program	0.523	M	Machine-type safety risk	0.468	M
ME4: Improper excavation and incremental launching program	0.431	M	Method-type safety risk	0.500	M
ME6: Improper soil conditioning program	0.523	M	Management-type safety risk	0.521	M
ME7: Improper grouting program	0.575	M	Environment-type safety risk	0.517	M
ME8: Sealed water-proof program	0.575	M	Geological environment safety risk	0.518	M
MN2: Safety institution;	0.523	M	The total onsite safety risk	0.501	M

As can be seen in Table 11, safety inspection (M5), safety organization & duty, quicksand layer (MN3), and high-pressure phreatic water (HE1) are at the high grade, which shows that these safety risks are more likely to cause safety accidents during shield construction. Thus, the onsite manager should develop a special construction plan in advance to identify and prevent these safety risks. Most of the safety risks are medium-grade risks and the results indicate that some common safety countermeasures should be taken to address these safety risks.

As for the different first-level safety risks, although they are all at the medium grade, the management-type safety risks have the maximum expected utility value, the environment-type safety risks, the personnel-type safety risks, and the method-type safety risks follow closely behind, and the machine-type safety risks have the minimum expected utility value. It indicates that non-standard management is still the most important factor causing safety accidents in China. Onsite managers should focus on the control of management-type, environment-type, and personnel-type safety risks. Besides, when compared to worker-type safety risks, manager-type safety risks have higher expected utility values, which indicates that managers are the core of safety risks. The design of safety institutions and management regulations should consider how to improve managers' safety management competency and how to motivate the managers' behavior intentions.

The total onsite safety risk is at the medium grade, which indicates that overall, the safety risk is at an average level. It also can be inferred that the project management department has an average level of management competency on safety risks during SSCUR. More safety experiences should be collected and more safety training should be carried out so that its manager can quickly and gradually improve control and emergency ability on shield construction safety risks.

## 6 Discussion and management implication

The article proposes a safety risk list of SSCUR, and the list includes five first-level safety risks and 38 second-level safety risks. This is the theoretical innovation of this study. The identified first-level safety risks are personnel-type, machine-type, method-type management-type, and environment-type safety risks, and the second-level safety risks were identified based on a literature review and expert interview. Compared to the prior studies, we identified more safety risks of subway shield construction when under-crossing a river. For instance, Chan et al. (62) investigated the very large tunnel safety risk across the Qiantang River, and identified machine-type, operation-type, and environment-type safety risks, but neglected the personnel-type and management-type safety risks. Some technique-focused papers only analyzed safety technology problems and the corresponding safety countermeasures when a subway crosses under a river (63–67). Thus, the safety risk list identified in this paper is more systematic and completed. It involves almost all subway construction safety risks when under-crossing a river, and has a stronger generalization ability, which can guide the shield construction practice when under-crossing a river.

This article proposed a new integrated approach to evaluate shield construction safety risk by integrating confirmatory factor analysis (CFA) and fuzzy evidence reasoning (FER). This is the method innovation of this study. CFA or SEM-kind methods have been introduced into safety risk assessment in the existing literature (68, 69), these methods can calculate the relationship degrees between some low-level variables (e.g., second-level safety risks) and an upper-level latent variable (e.g., first-level safety risk), and provide an approach for determining weights (56, 70). The advantages of these methods are that they make the weights of variables more objective by statistically analyzing large-scale questionnaire data. FER is also a widely used analytic technique in safety risk analysis (71, 72). This technique can partially resolve the semantic vagueness and uncertainty caused by an expert's evaluation and fuse multiple pieces of evidence to gain a comprehensive evidential inference (71, 73). Thus, compared to other evaluation models in shield construction safety risk, for instance, AHP, and FCE, the new proposed assessment model can gain more scientific and accurate results, which can provide a more precise foundation for safety risk prevention and controlling.

The proposed CFA-FER model was applied to a case, i.e., Zhengzhou subway line 12 under-crossing the Qili River, to test its feasibility. The calculation processes and results validate the proposed method can assess single safety risk and onsite total safety risk during shield construction under-crossing a river. The calculation results show that management-type safety risks have the maximum expected utility value. This finding is consistent with the prior literature and the actual situation in China. For instance, Zhang et al. (74) pointed out that management-type reasons are the most important ones causing safety accidents in China, non-standard safety management is still a common phenomenon in most project sites (75, 76). Besides, manager-type safety risk has a larger expected utility value than worker-type safety risk. This finding also can be explained based on prior research. As the core of the classic two-agent management model, the safety manager plays a leading role in safety management (77, 78). The workers' low safety awareness and high-frequency unsafe behaviors mainly derived from managers' negligence and the failure of the safety management system (9, 79, 80). Environment-type safety risks have relatively larger expected utility value, especially, the high-pressure phreatic water is the high-level safety risk. Previous literature has highlighted the significance of the hydrological environment for shield construction, and surface water and groundwater have been proven to be the direct causes of safety accidents such as excavation face collapse, water gushing, and even tunnel flooding (81, 82).

Based on the above-mentioned analysis, some management policies can be suggested for better on-site safety management. Firstly, management-type safety risks and manager-type safety risks were assessed to be the relatively higher safety risks. Senior and front-line managers can take the follow-up measures: (a) take China's indigenous management context and engineering reality into consideration when formulating management regulations; (b) establish a strict reward and punishment system, improve safety management systems, define managers' responsibilities and obligations, and strengthen the supervision of the construction process; (c) prioritize safety objective even if there are conflicts with schedule and cost objectives, and pay more efforts on the cultivation of high-level safety management personnel. Underground water and overburdened soil layers are the salient safety risks when shield construction crosses under a river. Some measures can also be conducted beforehand to mitigate the negative effect. Project managers can (a) further carry out in-depth geological and hydrological surveys; (b) grout in advance to reinforce the rich-water soil layer and ensure its stability before under-crossing; (c) control excavation speed and continuously monitor the water seepage of the tunnel.

## 7 Conclusion

The paper used a literature review and an expert interview to identify the safety risks of subway shield construction under-crossing a river, proposed a new safety risk assessment model by integrating CFA and FER, and employed a case to validate the feasibility of the proposed approach. The research conclusions are as follows:

(1) A practice-feasible safety risk list of shield construction under-crossing a river is identified, including 5 first-level safety risks and 38 second-level safety risks. The first-level safety risks include personnel-type, machine-type, method-type, environment-type, and management-type safety risks.

(2) An integrating safety risks assessment model was proposed to quantitatively assess the single safety risk and onsite total safety risk, and the model was validated feasible by applying it to a tunnel section of Zhengzhou subway line 12.

(3) A case study showed that safety inspection, safety organization and duty, quicksand layer, and high-pressure phreatic water were the high-level risks, the onsite total safety risk was at the medium level, and management-type safety risks, environment-type safety risks, and personnel-type safety risks have higher expected utility values. Besides, compared to worker-type safety risks, manager-type safety risks were expected to have higher risk-utility values.

(4) The paper only examined the safety risk assessment SSCUR by providing a safety risk list and an assessment approach. We did not analyze the correlation, causality, and coupling among safety risks, and examine how these safety risks cause safety accidents by mutual interaction. Follow-up studies can further investigate these questions.

## Data availability statement

The raw data supporting the conclusions of this article will be made available by the authors, without undue reservation.

## Ethics statement

Ethical review and approval was not required for the study on human participants in accordance with the local legislation and institutional requirements. Written informed consent from the participants was not required to participate in this study in accordance with the national legislation and the institutional requirements.

## Author contributions

KH: Conceptualization, Formal analysis, Writing – review & editing. TC: Investigation, Writing – original draft. JC: Project administration, Writing – review & editing. YH: Methodology, Writing – review & editing. HL: Validation, Writing – review & editing. HC: Validation, Writing – review & editing. KY: Resources, Writing – review & editing.
