# Supplementary material for: Safety risk assessment of subway shield construction under-crossing a river using CFA and FER
Source: Front Public Health. 2024 Feb 2;12:1279642. doi: 10.3389/fpubh.2024.1279642 (PMC10869540; doi:10.3389/fpubh.2024.1279642)
Supplement: Supplementary file 1 [file Table_1.pdf]

Appendix A The preliminary safety risk list

First-level safety risks		Second-level safety risks
Personnel-type safety risks	Worker-type safety risks	W1: Physical and psychological health; W2: Safety awareness; W3: safety skills; W4: safety experiences
	Manager-type safety risks	M1: Safety management awareness; M2: Safety management competency; M3: Safety management intention; M4: Safety communication; M5: Safety inspection; M6: Safety control.
Machine-type safety risks		MA1: Failure of cutter head system; MA2: Failure of thrust cylinder system; MA3: Failure of screw conveyor; MA4: Failure of segment erector; MA5: Failure of grouting system; MA6: Failure of ventilation system; MA7: Failure of soil transport vehicle; MA8: Failure of electrical equipment.
Method-type safety risks		ME1: Improper bank reinforcement program; ME2: Inadequate geological and hydrological investigation; ME3: Improper construction monitoring program; ME4: Improper excavation and incremental launching program; ME5: Improper center line control program; ME6: Improper soil conditioning program; ME7: Improper grouting program; ME8: Sealed water-proof program; ME9: Improper contingency plan.
Management-type safety risks		MN1: Safety culture; MN2: Safety institution; MN3: Safety climate; MN4: Safety organization & duty; MN5: Safety training & education; MN5: Safety management system.
Environment-type safety risks	Geological environment safety risks	GE1: Levee; GE2: Spur dike; GE3: Shallow overburden layer; GE4: Quick sand layer; GE5: Hydrogeological exploration borehole; GE6: Subterranean boulders; GE7: Subterranean voids.
	Hydrological environment safety risks	HE1: High-pressure phreatic water; HE2: High-pressure piestic water; HE3: High underground water level.
	Gaseous environment safety risks	GA1: Marsh gas (methane, hydrogen sulfide, etc.)

Appendix B The questionnaire for collecting data

**Safety risks**	**No importan t**	**Slightly importan t**	**Importan t**	**Relatively important**	**Extremely important**
W1: Physical and psychological health					
W2: Safety awareness					
W3: safety competency					
M1: Safety management awareness					
M2: Safety management competency					
M3: Safety management intentions					
M4: Safety communication					
M5: Safety inspection					
MA1: Failure of cutter head system					
MA2: Failure of thrust cylinder system					
MA3: Failure of screw conveyor					
MA4: Failure of segment erector					

MA5: Failure of grouting system					
MA6: Failure of ventilation system					
MA7: Failure of electrical equipment					
ME1: Improper bank reinforcement program					
ME2: Inadequate geological and hydrological investigation					
ME3: Improper construction monitoring program					
ME4: Improper excavation and incremental launching program					
ME5: Improper center line control program					
ME6: Improper soil conditioning program					
ME7: Improper grouting program					
ME8: Sealed water-proof program					
ME9: Improper contingency plan					
MN1: Safety culture					
MN2: Safety institution					
MN3: Safety organization & duty					
MN4: Safety training & education					
GE1: Levee;					
GE2: Spur dike					
GE3: Shallow overburden layer					
GE4: Quicksand layer					
GE5: Hydrogeological exploration borehole					
GE6: Subterranean boulders					
GE7: Subterranean voids					
HE1: High-pressure phreatic water					
HE2: High-pressure piestic water.					
GA1: Marsh gas (methane, hydrogen sulfide, etc.)					

Appendix C The demographic information of the 197 respondents.

Demographic variable	Category	Frequency	Percentage (%)
Gender	Male	142	72.1
	Female	55	27.9
Age	20-29 years	121	61.4
	30-39 years	46	23.4
	40-49 years	21	10.7
	50 years or more	9	4.6
Education degree	Associate college degree or below	77	39.1
	Bachelor's degree	91	46.2
	Master's degree or above	29	14.7
Tenure	0-5 years	112	56.9
	6-10 years	77	39.1
	More than 10 years	8	4.1
		14	7.1
Position	Project managers	14	7.1
	Department head	64	32.5

	Engineers	111	56.3
	others	8	4.1

Appendix D The safety risk checklist for experts' evaluation

Safety risks	Occurrence probability grade	Consequences severity grade
W1: Physical and psychological health		
W2: Safety awareness		
W3: safety competency		
M1: Safety management awareness		
M2: Safety management competency		
M3: Safety management intentions		
M4: Safety communication		
M5: Safety inspection		
MA1: Failure of cutter head system		
MA2: Failure of thrust cylinder system		
MA3: Failure of screw conveyor		
MA4: Failure of segment erector		
MA5: Failure of grouting system		
MA6: Failure of ventilation system		
MA7: Failure of electrical equipment		
ME1: Improper bank reinforcement program		
ME2: Inadequate geological and hydrological investigation		
ME3: Improper construction monitoring program		
ME4: Improper excavation and incremental launching program		
ME5: Improper center line control program		
ME6: Improper soil conditioning program		
ME7: Improper grouting program		
ME8: Sealed water-proof program		
ME9: Improper contingency plan		
MN1: Safety culture		
MN2: Safety institution		
MN3: Safety organization & duty		
MN4: Safety training & education		
GE1: Levee		
GE2: Spur dike		
GE3: Shallow overburden layer		
GE4: Quicksand layer		
GE5: Hydrogeological exploration borehole		
GE6: Subterranean boulders		
GE7: Subterranean voids		
HE1: High-pressure phreatic water		
HE2: High-pressure piestic water		
GA1: Marsh gas (methane, hydrogen sulfide, etc.)		
